# Thank you to our reviewers of 2024

**DOI:** 10.1002/epi4.13129

**Published:** 2025-02-07

The Editors of *Epilepsia Open* extend their heartfelt gratitude to our Associate Editors, editorial board members, and the following individuals for their invaluable contributions in reviewing manuscripts over the past year. Your insight, dedication, and generous investment of time and expertise have been essential to our mission. We deeply appreciate your efforts in supporting the advancement of knowledge and the success of our journal.

Merab Kokaia , PhD

Editor‐in‐Chief, *Epilepsia* *Open*

merab.kokaia@med.lu.se

Piero Perucca , MD, PhD

Deputy Editor, *Epilepsia* *Open*

Aristea S. Galanopoulou, MD, PhD

Past Editor‐in‐Chief, *Epilepsia* *Open*

Dong Zhou, MD

Past Deputy Editor, *Epilepsia* *Open*

***≥6 reviews, **≥3–5 reviews, *1–2 reviews

Stéphane Auvin***

Jaime Carrizosa***

Giulia Sofia Cereda***

Bruce Hermann***

Alexandra Klotz***

Eric Kossoff***

Simona Lattanzi***

Paolo Mainardi***

David McArthur***

Angelo Pascarella***

Ramya Raghupathi***

Nicola Specchio***

Kette Valente***

Pedro Viana***

Johan Zelano***

Action Amos**

Tobias Baumgartner**

Massimiliano Beghi**

Vincenzo Belcastro**

Christophe Bernard**

Valentina Biagioli**

Francesca Bisulli**

Christian Brandt**

Silvia Buratti**

Alessandro Consales**

Arthur Cukiert**

Luca De Palma**

Gianluca D'Onofrio**

Camilo Espinosa‐Jovel**

Giovanni Falcicchio**

Edward Faught**

Edoardo Ferlazzo**

Alessandro Ferretti**

Emma Foster**

Marian Galovic**

Elena Gardella**

Aurelie Hanin**

Xiaosong He**

Shaun Hussain**

Charuta Joshi**

Dorothee Kasteleijn‐Nolst Trenite**

Elisabeth Kaufmann**

Mark Keezer**

Miriam Kessi**

Jochen Klein**

Harley Kurata**

Naoto Kuroda**

Gaetan Lesca**

Kheng Seang Lim**

Stefano Meletti**

Cameron Metcalf**

Mathieu Milh**

Solomon Moshe**

Justyna Paprocka**

Prachi Parikh**

Phillip Pearl**

Roro Rukmi Windi Perdani**

Tommaso Pippucci**

Francesco Pisani**

Ronit Pressler**

Vineet Punia**

Isha Puntabekar**

Georgia Ramantani**

Christopher Reid**

Antonella Riva**

Zhichao Ruan**

Guido Rubboli**

Lucas Sainburg**

Josemir Sander**

Daniel San‐Juan**

Marcello Scala**

Andreas Schulze‐Bonhage**

Jo Sourbron**

Adam Strzelczyk**

Kenji Sugai**

Soumya Sundaram**

Christopher Tailby**

Tracie Tan**

Venus Tang**

Naotaka Usui**

Lata Vadlamudi**

Andreas van Baalen**

Alberto Verrotti di Pianella**

Greta Volpedo**

Janelle Wagner**

Elaine Wirrell**

Huifang Yan**

Gaetano Zaccara**

Ifrah Zawar**

Daniel Zhou**

Taylor Abel*

Karen Joy Adiao*

Naoki Akamatsu*

Alawi Alattas*

Demet Algın*

Amza Ali*

Joseph Anderson*

Saadet Andrews*

Johan Arends*

Caren Armstrong*

Alexis Arzimanoglou*

Melody Asukile*

David Auerbach*

Jerome Aupy*

Jerome Badaut*

Valeria Badioni*

Irene Bagnasco*

Mark Baker*

Ganna Balagura*

Sara Baldassari*

Simona Balestrini*

Alice Ballabeni*

Alice Ballerini*

Carmen Barba*

Emmanuel Barbier*

Domenica Battaglia*

Stéphanie Baulac*

Kristie Bauman*

Marcel Bausch*

Allan Bayat*

E. Martina Bebin*

Albert Becker*

Christoph Beier*

Abebe Muche Belete*

Elinor Ben‐Menachem*

Mark Bennett*

Sophie Bennett*

Federica Bertaso*

Frank Besag*

Sonam Bhalla*

Elisa Bianchi*

Leonilda Bilo*

Johan Bjellvi*

Varina Boerwinkle*

Thomas Bonduelle*

Josh Bonkowsky*

Valeri Borger*

Karin Borges*

Ingo Borggrafe*

Luca Bosisio*

Stefania Bova*

Geraldine Boylan*

Cristina Bravo*

Amy Brewster*

Stephen Briggs*

Eva Brilstra*

Walter Bruchhausen*

Andreas Brunklaus*

Jeffrey Buchhalter*

Donna Cabalo

Shuying Cai*

Jillian Cameron*

Dezhi Cao*

Roberto Caraballo*

Patrick Carney*

Marco Carotenuto*

Josephine Casanova‐Gutierrez*

James Castellano*

Mackenzie Cervenka*

Felix Chan*

Kevin Chapman*

Nicole Chemaly*

Cong Chen

Lei Chen*

Kuo‐Liang Chiang*

Richard Chin*

Yotin Chinvarun*

Catherine Christian‐Hinman*

Bahar Çiftçi*

Erica Cognolato*

Francesca Colciaghi*

Kelly Conner*

Edward Cooper*

Daniëlle Copmans*

Antonietta Coppola*

Marie‐Constance Corsi*

Massimo Cossu*

Daniel Costello*

Claudia Cuccurullo*

Linda Dalic*

David Darrow*

Sandhitsu Das*

Alexandre Datta*

Marco de Curtis*

Matthias De Wachter*

Ahmad Reza Dehpour*

Ennio Del Giudice*

Giovanni Battista Dell'Isola*

Nele Demeyere*

Antoine Depaulis*

Judith Dexheimer*

Robertino Di Lena*

Gianluca Dini*

Elena Dossi*

Mattias Duempelmann*

Gian Marco Duma*

Danielle Eekers*

Maurizio Elia*

Andrew Escayg*

Alina Esterhuizen*

Antonio Falace*

Merce Falip*

Raffaele Falsaperla*

Romano Ferruccio*

Martha Feucht*

Melissa Filippini*

Büşra Filiz Genç*

Robert Fisher*

Martine Fohlen*

Michael Fong*

Jonah Fox*

Jacqueline French*

Daniel Friedman*

Mayu Fujikawa*

Lucia Fusco*

Kais Gadhoumi*

Antonio Gambardella*

Rimma Gamirova*

Rita Garbelli*

Divyani Garg*

Tyler Gaston*

Enguday Gebeyehu*

Bekalu Getachew*

Fatemeh Ghaffari*

Taha Gholipour*

Tania Giangregorio*

Gian Luigi Gigli*

Giada Giovannini*

Giorgia Giussani*

Vadym Gnatkovsky*

Zachary Goldblum*

Daniel Goldenholz*

Sam Gooley*

Jean Gotman*

Soundarya Gowda*

Philip Grewe*

James Gugger*

Jianxiong Gui*

MV Gule*

Hiba Haider*

Amy Halliday*

Marla Hamberger*

Kevin Hampel*

Chellamani Harini*

Hans Hartmann*

Christoph Helmstaedter*

David Henshall*

Michael Hildebrand*

Martin Hirsch*

Manisha Holmes*

Annette Holth Skogan*

Martin Holtkamp*

Fengling Hu*

Rod Hunt*

Akio Ikeda*

Imran Imran*

Sara Inati*

Yushi Inoue*

Floor Jansen*

Prashant Jauhari*

Jianxiong Jiang*

Wen Jiang*

Yuwu Jiang*

Jin Jing*

Erica Johnson*

Meredith Johnson*

Nigel Jones*

Holger Joswig*

Shilpa Kadam*

Marios Kaliakatsos*

Gautam Kamila*

Peter Kaplan*

Ioannis Karakis*

Philippa Karoly*

Ayşe Kartal*

Dimitrios Kazis*

Sarah Kelley*

Elaine Kiriakopoulos*

Najib Kissani*

Johanna Kissler*

Gerhard Kluger*

Katsuhiro Kobayashi*

Sookyong Koh*

Günter Krämer*

Ruzica Kravljanac*

Heinz Krestel*

Izumi Kuramochi*

Angelo Labate*

Lieven Lagae*

Ilaria Lagorio*

Suncica Lah*

Jacopo Lanzone*

Antonio Leo*

Ilo Leppik*

Qiang Li*

Tingsong Li*

Jianxiang Liao*

Mark Libenson*

Yicong Lin*

Zehong Lin*

Chang Liu*

Ling Liu*

Qing Liu*

Lili Long*

Iscia Lopes‐Cendes*

Wolfgang Löscher*

Katarzyna Lukasiuk*

Huaxia Luo*

Yanli Ma*

Emma Macdonald‐Laurs*

Lufuno Makhado*

Mahdi Malekpour*

Masa Malenica*

Federica Malerba*

Stephen Malone*

Mark Manford*

Matteo Mangoni*

Massimo Mantegazza*

Xiao Mao*

Giorgi Margvelani*

Oreste Marsico*

Benoît Martin*

Christopher Martinez Aguirre*

Federico Mason*

Massimo Mastrangelo*

Joyce Matsumoto*

Thomas Mayer*

Luis Mayor‐Romero*

Andrey Mazarati*

Cian McCafferty*

John McGinley*

Oriano Mecarelli*

Marco Medina*

Davide Mei*

Daniel Molla Melese*

Francesco Miceli*

Janet Mifsud*

Mohamad Mikati*

Laurie Miller*

James Mills*

Nishant Mishra*

Zarine Mogal*

Sara Mole*

Patrick Moloney*

Tonmoy Monsoor*

Qingxin Mu *

Lorenzo Muccioli*

Jagarlapudi Murthy*

Kenneth Myers*

Maryam Nabavi Nouri*

Nael Nadif Kasri*

Nobukazu Nakasato*

Hiroki Nariai*

Georgios Naros*

Oliver Neal*

Marcus Ng*

Lena Nguyen*

John‐Paul Nicolo*

Russell Nightscales*

Annacarmen Nilo*

Takuji Nishida*

Daniele Noel*

Douglas Nordli III*

Amani M. Norling*

Georgios Ntolkeras*

Hirokazu Oguni*

Heather Olson*

Sandra Orozco‐Suarez*

Alessandro Orsini*

Cigdem Ozkara*

Juan Fernando Padin Nogueira*

Trudy Pang*

Heath Pardoe*

Pasquale Parisi*

Kristen Park*

Manisha Patel*

Piero Pavone*

Giovanni Pellegrino*

Jacob Pellinen*

Florence Perrin*

M. Scott Perry*

Marco Perulli*

Elia Pestana‐Knight*

YH Phaust*

Luciana Pimentel‐Silva*

Laura Rosa Pisani*

Asla Pitkänen*

Julia Plank*

Tilman Polster*

Amber Postma*

Ofer Prager*

Suresh Pujar*

Claudio Queiroz*

Harikumar Rajaguru*

Martin Rakusa*

Stefan Rampp*

Doodipala Reddy*

Jan Rémi*

Anny Reyes*

Jong Rho*

Joshua Richman*

Marian Roan*

Eleonora Rosati*

Alexander Rotenberg*

Jessica Royer*

Theodor Rüber*

Gabriele Ruffolo*

Justin Ryan*

Arushi Saini*

Iván Sánchez Fernández*

Raman Sankar*

Andrea Santangelo*

Vedat Sar*

Rani Sarkis*

Harvey Sarnat*

Silvana Sarria‐Estrada*

Abhishek Sathe*

Morris Scantlebury*

Frederic Schaper*

Kai Michael Schubert*

Rod Scott*

GianPietro Sechi*

Franck Semah*

Yonatan Serlin*

Pedro Serrano‐Castro*

Eilon Shany*

Suvasini Sharma*

Renée Shellhaas*

Hideaki Shiraishi*

Young‐Min Shon*

Pallop Siewchaisakul*

Timothy Simeone*

Michele Simonato*

Nishant Sinha*

Saurabh Sinha*

Mary Lou Smith*

Michael Smith*

Stuart Smith*

Daichi Sone*

Vincenzo Sortino*

Cesar Soutullo*

Ashwini Sri Hari*

Wiliam Stacey*

Carl Stafstrom*

Catherine Stamoulis*

Donnie Starnes*

Alena Stasneko*

Hermann Stefan*

Mirja Steinbrenner*

Claude Steriade*

Tommy Stödberg*

Pasquale Striano*

Hidenori Sugano*

Olga Sukocheva*

Joseph Sullivan*

Joseph Symonds*

Charles Szabo*

Jerzy Szaflarski*

Alan Talevi*

Xinjie Tan*

Tomotaka Tanaka*

James Tao*

Laura Tassi*

Dimitris N. Tatakis*

César Teixeira*

Mekonnen Tesfaw*

Roland Thijs*

Liu Lin Thio*

Maoqiang Tian*

Xiaojuan Tian*

Xin Tian*

Steven Tobochnik*

Jun Tohyama*

Itay Tokatly Latzer*

Manuel Toledo‐Argany*

Joseph Tracy*

Chahnez Triki*

Manjari Tripathi*

Francesco Turco*

Mitsugu Uematsu*

Aycan Ünalp*

Pieter Van Mierlo*

Anna Vaudano*

Libor Velisek*

Fabiana Vercellino*

Richard Verrier*

Gianmichele Villano*

Vicente Villanueva*

Laurent Villard*

Kollencheri Puthenveettil Vinayan*

Berthold Voges*

Christian Vollmar*

Felix von Podewils*

Randi von Wrede*

Konrad Wagstyl*

Britta Wandschneider*

Mengyang Wang*

Shuang Wang*

Xuefeng Wang*

Yu Wang*

Amanda Saphira Wardani*

Sarah Weckhuysen*

Benjamin Whatley*

Robyn Whitney*

Leah Wibecan*

Jo Wilmshurst*

Yaroslav Winter*

Juri‐Alexander Witt*

Stefan Wolking*

Sheng Wong*

Lily Wong‐Kisiel*

David Wright*

Ye Wu*

Han Xie*

Cenglin Xu*

Rezzan Yildiz*

Tulay Yılmaz Erol*

Elissa Yozawitz*

Aleksey Zaitsev*

Jianzhao Zhang*

Jie Zhang*

Yuehua Zhang*

Xiu‐He Zhao*

Zhihong Zhuo*

Maeike Zijlmans*

Janneke Zinkstok*

